# Associated factors of burnout among Chinese vaccination staff during COVID-19 epidemic: A cross-sectional study

**DOI:** 10.3389/fpubh.2023.1086889

**Published:** 2023-03-08

**Authors:** Wenwen Gu, Yan Liu, Zhaojun Lu, Jun Wang, Xinren Che, Yuyang Xu, Xuechao Zhang, Jing Wang, Jian Du, Xiaoping Zhang, Junfang Chen

**Affiliations:** Department of Immunization and Prevention, Hangzhou Center for Disease Control and Prevention, Hangzhou, China

**Keywords:** prevalence, vaccination staff, COVID-19, burnout, China

## Abstract

**Objective:**

During the COVID-19 epidemic, vaccination staff had three main aspects of work: routine vaccination for children and adults, COVID-19 vaccination and COVID-19 prevention and control. All these works significantly increased the workload of vaccination staff. This study aimed to investigate the prevalence and influencing factors of burnout among vaccination staff in Hangzhou, China.

**Methods:**

A total of 501 vaccination staff from 201 community/township healthcare centers in Hangzhou were recruited using a cross-sectional survey through WeChat social platform. The Maslach Burnout Inventory-General Scale (MBI-GS) was used to assess the level of burnout. Descriptive statistics were made on the characteristics of participants. Univariate analysis using the chi-square test and multivariable analysis using binary logistic regression were conducted to determine the relative predictors of burnout. Univariate analysis and multiple linear regression were used to determine the relative predictors of exhaustive emotion, cynicism, and personal accomplishment.

**Results:**

During the COVID-19 pandemic, 20.8% of the vaccination staff experienced burnout. Educational level above undergraduate education level, medium professional title, and more working time in COVID-19 vaccination work reported a higher degree of job burnout. The vaccination staff was experiencing a high degree of exhaustive emotion, cynicism, and low personal accomplishment. Professional title, working place, and working time for COVID-19 vaccination were associated with exhaustive emotion and cynicism. Professional title and participation time for COVID-19 prevention and control were associated with personal accomplishment.

**Conclusions:**

Our findings suggest that the prevalence rate of burnout is high among vaccination staff during the COVID-19 pandemic, especially with a low level of personal accomplishment. Psychological intervention for vaccination staff is urgently needed.

## 1. Introduction

An unprecedented outbreak of pneumonia of unknown etiology in Wuhan, Hubei Province, China, emerged in December 2019 (1, 2). It was named coronavirus disease 2019 (COVID-19) caused by Severe Acute Respiratory Syndrome Coronavirus type 2 (SARS-CoV-2) (3). On March 11, 2020, the World Health Organization (WHO) officially classified the global COVID-19 outbreak as a pandemic (4). Although countries worldwide have taken active and effective measures to control the epidemic, the current global epidemic is still severe (5). As of September 22, 2022, 610 million confirmed cases and 6.5 million deaths had been reported globally (6).

Since the outbreak, China, one of the countries with the most severe COVID-19 epidemic in the world, has implemented several strict but effective measures, such as lockdown cities, controlling traffic (7), mass isolation of individuals with cases (8), construction of Fangcang shelter hospitals (9), and public education campaigns encouraging the use of masks and hand washing (10). One of the most effective measures to prevent COVID-19 was COVID-19 vaccination. The COVID-19 vaccine is remarkably effective in preventing severe COVID-19 symptoms and death, and the COVID-19 booster vaccination can further improve the protective effect. Studies found that the risk of developing severe COVID-19 disease for those aged 18 to 59 who had received a booster COVID-19 vaccination was 94% lower than those who did not receive the vaccine. For people 60 and older, the figure is 95% (11). Since July 2020, China has officially launched emergency vaccination for high-risk exposed groups, including frontline medical workers, border and port staff, et al. In December 2020, the vaccination of key population groups, including cold chain logistics staff, medical staff, public transport workers, et al., was launched. Since then, China has gradually expanded the age range for COVID-19 vaccination from 18 years old and above to 3 years old and above. Currently, the inactivated COVID-19 vaccine in China is administered in three doses (12–14).

Vaccination staff at community/township health service centers are the leading force in COVID-19 vaccination in Hangzhou, China. Vaccination staff refers to all the personnel working in the vaccination clinic, including health prechecker, registration personnel, inoculator, logistics manager, etc. According to “Technical specifications for vaccination work” issued by the National Health Commission (15), each town (subdistrict) has a vaccination clinic set up in the community/township health service center. Before the COVID-19 epidemic, the vaccination staff was primarily responsible for childhood vaccination, including Expanded Program on Immunization (EPI) and Non-Expanded Program on Immunization (non-EPI) vaccination, as well as adult vaccination, such as flu vaccine, HPV vaccine, 23-valent pneumonia vaccine, and herpes zoster vaccine, et al. Their work included vaccination, cold chain management, adverse events following immunization (AEFI) reporting, report form filling, vaccine education, and other works. During the COVID-19 pandemic, the job of COVID-19 vaccination had fallen to them. Vaccine recipients are expanded from children and a few adults to the entire population over the age of three. In addition, vaccination staff, as primary care workers, also work on COVID-19 prevention and control, including nucleic acid sampling, elimination, hospital transmission, et al. (16). All these works significantly increased the working hours and workload of the vaccination staff.

According to previous studies, the epidemic of COVID-19 had placed a severe strain on healthcare workers (17–20) and significantly increased psychological problems of job burnout (20). As first described by Freudenberger (21), and subsequently developed by Maslach and Leiter (22) and Maslach et al. (23), chronic stress associated with emotionally intense work demands for which resources are inadequate can result in burnout. The three critical dimensions of this response are overwhelming exhaustion, feminism and detachment from the job, and a sense of ineffectiveness and lack of accomplishment (24). The exhaustion dimension is also described as wearing out, losing enerlosingletion, debilitation, and fatigue. The cynicism dimension was originally called depersonalization (given the nature of human services occupations), but is also described as negative or inappropriate attitudes toward clients, irritability, loss of idealism, and withdrawal. The inefficacy dimension was originally called reduced personal accomplishment and is also described as reduced productivity or capability, low morale, and an inability to cope (24). The first burnout measure based on a comprehensive program of psychometric research was the Maslach Burnout Inventory (MBI). It has been considered the standard tool for research in this field and has been translated and validated in many languages.

Prior studies showed that job burnout was high among medical staff during COVID-19. A survey has reported that 34.7% of physicians suffer from job burnout in Canada (25). In Huo et al. (11) study, about 34.5% of medical staff experienced burnout. For nurses, a study showed that about half of the nurses reported moderate and high work burnout in China (26). It is worth noting that job burnout could have many negative consequences. In terms of work, burnout is frequently associated with various forms of negative reactions and job withdrawal, including job dissatisfaction, low organizational commitment, absenteeism, turnover, lower productivity, and impaired quality of work (27–30). In addition, burnout can be “contagious” (31, 32). It could have a negative impact on colleagues, both by causing more significant personal conflict and by disrupting job tasks. In terms of personal health, burnout could contribute to poor health, which in turn contributes to burnout (33).

Vaccination staff plays an essential role in preventing and controlling the COVID-19 epidemic. They are responsible for routine and COVID-19 vaccination and, meanwhile, like other primary health care workers, for COVID-19 prevention and control. Currently, the COVID-19 pandemic continues to be a global threat, and SARS-CoV-2 is still developing (34). In the future, vaccination with a booster shot of the COVID-19 vaccine is still an important measure to prevent COVID-19 (35). Mass vaccination of the whole population will likely become routine work. Therefore, it is crucial to determine the influencing factors of job burnout of vaccination staff and reduce their job burnout.

There were many studies on job burnout in different medical specialties, such as nurses, doctors, physicians. No studies on burnout among vaccination staff have been found. This paper filled this gap in the literature by providing an in-depth exploration of the mental health of vaccination staff during the COVID-19 pandemic. This study attempted to gain a deeper understand of this reality and to contribute as much as possible to this important group of vaccination staffs in pandemic. The findings not only offered a scientific foundation for group intervention research involving vaccination staff, but also provided scientific basis for further strengthening the vaccination campaign during the COVID-19 pandemic, and could be a reference for job burnout of vaccination staff in other regions of China.

## 2. Materials and methods

### 2.1. Study design and participants

We conducted a cross-sectional survey to assess the job burnout of vaccination staff in Hangzhou, China, during the COVID-19 epidemic from June 10 to 17. Hangzhou, the capital city of Zhejiang Province, is a well-developed city in eastern China. Under the jurisdiction of the City of Hangzhou are 10 urban districts, one county-level city, two counties, and a total of 191 towns (subdistricts). By the end of 2021, Hangzhou's permanent residents population totals 12.204 million (36). Hangzhou had few cases of COVID-19 before 2022, and all were imported cases. Since the beginning of 2022, locally transmitted confirmed cases of COVID-19 emerged in Hangzhou, and several cluster infections occurred. Hangzhou doubled down on efforts to prevention and control the epidemics. According to the above reasons, the information collection in this survey starts in January 2022. To calculate the sample size for this survey, we referred to previous literature (19) and hypothesized that 30% of vaccination staff would have a level of burnout at a margin of error ± 6%, and we assumed a 95% confidence interval, a power of 80%. Using a sample size calculator and considering 14 factors to be entered in the multivariable analysis, the target sample size was 457. Then we added a 10% non-respondent rate, giving a final sample size of 500. To avoid face-to-face interaction, we edited the questionnaire on the Wen Juan Xing online platform, formed a link to the questionnaire, and sent it to each survey respondent *via* WeChat, one of mainland China's most essential and widely used social tools. The respondents answered the self-administered questionnaire by visiting the Uniform Resource Location (URL) on their phones. All 201 vaccination clinics in Hangzhou participated in the survey, and at least two vaccination staff were randomly selected from each clinic to participate in the survey. Finally, a total of 501 vaccination staff were recruited. All the participants were given consent to participate and assured de-identification and confidentiality in handling their data before they answered the questionnaires.

The studies involving human participants were reviewed and approved by the Ethics Committee of the Hangzhou municipal center for disease control and prevention. The participants provided their *written* informed consent to participate in this study.

### 2.2. Assessments tools

#### 2.2.1. Assessment of socio-demographic and work-related factors

A self-administered questionnaire was designed to collect socio-demographic information. The following socio-demographic factors were assessed: gender (male/female), age, marital status (currently married, currently not married), education level (less than undergraduate, undergraduate and above), family income (< 5,000 CYN, 5,000–9,999 CYN, 10,000–19,999 CYN, 20,000–29,999 CYN, ≧30,000 CYN), working years, professional title (junior, medium, senior), working place (urban, suburb, rural).

We divided the work of vaccination staff during the epidemic of COVID-19 into three main categories: routine vaccination work, COVID-19 vaccination work, and COVID-19 control, and prevention work. Variables of routine vaccination work included the daily number of vaccinations in each vaccination clinic (< 100 persons, 100–199 persons, 200–299 persons, ≧300 persons), weekly vaccination working days for each vaccination clinic (0.5 days, 1 day, 1.5–2 days, 2.5–3 days, ≧3 days). Variables of COVID-19 vaccination work included the doses of COVID-19 vaccination in each vaccination clinic in 2022 (0–9,999 doses, 10,000–19,999 doses, 20,000–39,999 doses, 40,000–59,999 doses, ≧60,000 doses), the extent to which COVID-19 vaccination work takes up time off work or rest days (not participating, during working time, a few of works take up time off work or rest days, most of work takes up time off work or rest days, all the work takes up time off work or rest days). Variables of COVID-19 prevention and control work included the extent to which COVID-19 prevention and control work takes up time off work or rest days (not participating, during working time, a few of works take up time off work or rest days, most of work takes up time off work or rest days, all the work takes up time off work or rest days), and duration of participation in COVID-19 prevention and control work (not participating, < 1 week, 1 week-1month, 1-2 months, ≧2 months).

#### 2.2.2. Assessments for burnout

The Chinese version of the Maslach Burnout Inventory General Survey (MBI-GS) (37) was used to assess job burnout in this survey, which has been widely used among healthcare workers in China. MBI-GS consists of three dimensions of job burnout: Emotional Exhaustion (EE): (5 items), which means feelings of being emotionally overextended and depleted of one's emotional resources; Cynicism (CY) (4 items), which means a negative, callous, or excessively detached response to other people; Personal Accomplishment (PA): (6 items), which means a decline in one's feelings of competence and achievement in one's work. Each item consists of a 7-point Likert scale: 0 = never, 1 = barely, 2 = occasionally, 3 = often, 4 = frequently, 5 = very frequently, and 6 = every day, ranging from 0 (“never”) to 6 (“every day”). Higher scores on the dimensions of EE and CY indicate burnout, and so as the lower scores on the dimension of PA. The MBI-GS has shown good reliability and validity in previous studies in China (38, 39). In this study, the result of reliability analysis showed that the scale was in a high level of internal consistency in all three dimensions in the current sample. The Cronbach's alpha for all 15 items was 0.900, and for EE, CY and PA was 0.963, 0.942, and 0.936, respectively.

Based on several previous studies in China (19, 40), subscales scores are considered as low, moderate, or high level of burnout syndrome according to these cut-points: low EE < 9, moderate EE 9–13, high EE>13; low CY < 3, moderate CY 3–9, high CY>9; low PA < 18, moderate PA 30–18, high PA>30. High EE and high CY or low PA are conditions for burnout (“exhaustion+1”), which is considered to be the most effective categorization to distinguish between individuals with high and low burnout (41).

### 2.3. Statistical analysis

Frequencies and percentages were summarized for the categorical variables. Mean and standard deviation (SD) were calculated for continuous numerical data. Comparisons of sociodemographic and work-related variables of participants between the burnout group and the non-burnout group were analyzed by chi-square test. A multivariable analysis using binary logistic regression was conducted to determine the relative predictors of burnout when controlled for potential confounding among the various predictor variables. Correlates with a *P* < 0.1 in the univariate analysis were included in the multivariable analysis using the “Forward: LR” method. Then, to further identify the independent factors associated with MBI-GS scores, variables with *P* < 0.1 in the univariate analysis were entered into the multiple linear regression, with the MBI-GS subscores as dependent variables. All statistical analyses were conducted using SPSS (version 24.0).

## 3. Results

### 3.1. Demographic characteristics and work-related situations of participants

In total, 501 individuals were included in the analysis. Among all the participants, 85.2% were female, and 14.8% were male. Almost half of the participants were in the age range of 31–40 (50.5%). 50.9% had a junior professional title. The majority of participants were married (82.6%), undergraduate and above (73.7%), and had household incomes between 5,000–9,999 CNY (40.9%) and 10,000–19,999 CNY (32.1%). The average working years was 14.03 ± 8.1 years. 36.3% of the participants worked in urban areas, 42.1% in suburbs, and 36.3% in rural areas. Most participants held two or more jobs at the same time. Majority of participants were responsible for registration (73.1%), followed by inoculation (61.3%) and health pre-check (56.1%). Other jobs (5.2%) included report filling, administration, etc.

Regarding routine vaccination work, 47.3% of the participants worked in vaccination clinics with a daily number of vaccinations < 100 people. 36.3 and 31.7% of participants worked in the vaccination clinic with 1 day per week and 2.5–3 days per week vaccination working time, respectively.

In terms of COVID-19 vaccination work, 27.9% of the participants worked in vaccination clinics that had administered 0–9,999 doses of COVID-19 vaccine, and the proportion administering 10,000–19,999 doses, 20,000–39,999 doses, 40,000–59,999 doses, and ≧60,000 doses were 25.1, 17.0, 12.4, and 17.6%, respectively. For COVID-19 vaccination working time, more than half of the participants (59.7%) reported that few works took up time off work or rest days.

In terms of COVID-19 prevention and control work, more than half of participants (51.9%) indicated that few works took up time off work or rest days. 56.3% of participants had been involved in this work for over 2 months.

More detailed information about participants' demographic and job-related characteristics is shown in Table 1.

**Table 1:** Social-demographic and work-related situations of participants.

**Variables**		***N***	**%**
**Sociodemographic characteristics**			
Age	18–30	107	23.4
	31–40	253	50.5
	41–50	104	20.8
	>50	27	5.4
Gender	Men	74	14.8
	Women	427	85.2
Marriage status	Currently married	414	82.6
	Currently not married	87	17.4
Education level	< Undergraduate	132	26.3
	≧Undergraduate	369	73.7
Family income	< 5,000 CYN	48	9.6
	5,000–9,999 CYN	205	40.9
	10,000–19,999 CYN	161	32.1
	20,000–29,999 CYN	45	9.0
	≧30,000 CYN	42	8.4
Working years	(Mean ± SD)	14.03 (8.1)	
Professional title	Junior	255	50.9
	Medium	216	43.1
	Senior	30	6.0
Working place	Urban	108	21.6
	Suburb	211	42.1
	Rural	182	36.3
Occupational classification	Health precheck	281	56.1
	Registration	366	73.1
	Inoculation	307	61.3
	Health observation after inoculation	135	26.9
	Logistics management	184	36.7
	Others	26	5.2
**Daily vaccination work**			
Daily number of vaccinations^a^	< 100 persons	237	47.3
	100–199 persons	201	40.1
	200–299 persons	55	11.0
	≧300 persons	8	1.6
Vaccination working days per week	0.5 day	41	81.8
	1 day	182	36.3
	1.5–2 days	74	14.8
	2.5–3 days	159	31.7
	≧3.5 days	45	9.0
**COVID-19 vaccination work**			
COVID-19 vaccination doses^b^	0–9,999 doses	140	27.9
	10,000–19,999 doses	126	25.1
	20,000–39,999 doses	85	17.0
	40,000–59,999 doses	62	12.4
	≧60,000 doses	88	17.6
Working time	Not participating	30	5.9
	During working hours	72	14.4
	A few of works take up time off work or rest days	299	59.7
	Most of work takes up time off work or rest days	82	16.4
	All the work takes up time off work or rest days	18	3.6
**COVID-19 prevention and control work**			
Working time	Not participating	23	4.6
	During working hours	45	9.0
	A few of works take up time off work or rest days	260	51.9
	Most of work takes up time off work or rest days	149	29.7
	All the work takes up time off work or rest days	24	4.8
Participation time	Not participating	23	4.6
	< 1 week	20	4.0
	1 week−1 month	69	13.8
	1–2 months	107	21.4
	≧2 months	282	56.3

a : Daily number of vaccinations for routine vaccines in each vaccination clinic.

b : The doses of COVID-19 vaccination in each vaccination clinic in 2022.

**Figure 1:**
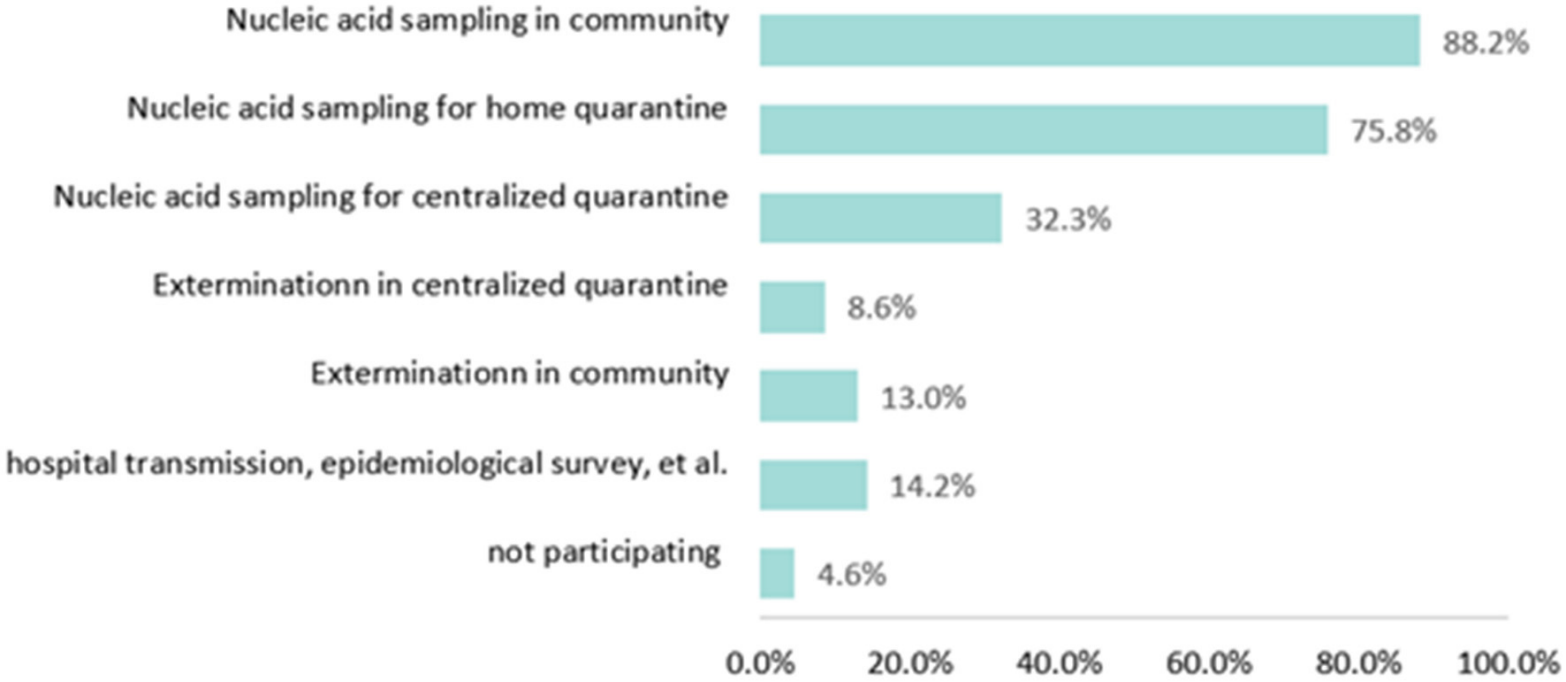
Distribution of different types of COVID-19 prevention and control work.

As shown in Figure 1, the majority of the vaccination staff participated in nucleic acid sampling work in the community (88.2%) and nucleic acid sampling work for home quarantine (75.7%). About one-third of vaccination staff (32.3%) participated in nucleic acid sampling for centralized quarantine. 14.2% of vaccination staff participated in other prevention and control work for COVID-19, including nucleic acid sampling at highway chokepoints, epidemiological investigation of close contacts, hospital transmission, et al. (Figure 1).

### 3.2. Prevalence of burnout in vaccination staff

**Figure 2:**
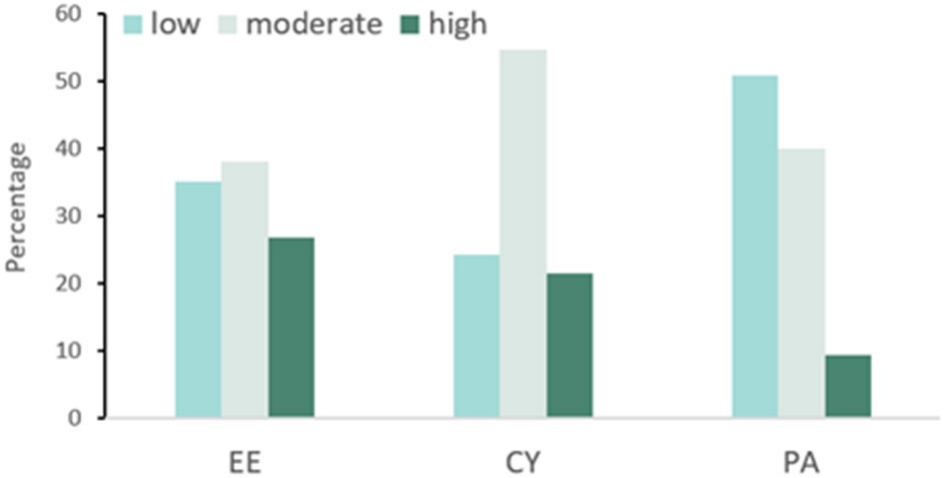
Distribution of different severity levels in different dimensions in burnout.

The prevalence of burnout in vaccination staff was 20.8% (104/501). For EE, 26.7% (134/501), 38.1% (191/501), and 35.1% (176/501) vaccination staff were at a high, moderate, and low level, respectively. For CY, high, moderate, and low levels accounted for 21.4% (107/501), 54.5% (273/501), and 24.2% (121/501), respectively. For PA, almost half of the vaccination staff were at a low level (50.7%, 254/501), 39.9% (200/501), and 9.4% (47/501) were at a moderate and a high level (Figure 2).

### 3.3. Factors associated with burnout

Chi-squared tests revealed that there were significant differences between burnout and non-burnout groups in terms of age, education level, professional title, working place, the daily number of vaccinations, COVID-19 vaccination doses, working time of COVID-19 vaccination, working time and participation time of COVID-19 prevention and control (all *P* < 0.1). The burnout rates of each type of variable are shown in Table 2.

**Table 2:** Univariate analysis of the associated factors of burnout among vaccination staff.

**Variables**		**No burnout**		**Burnout**		***P***
		***N***	**%**	***N***	**%**	
**Sociodemographic characteristics**						
Age	18–30	99	84.6%	18	15.4%	**0.009**
	31–40	192	75.9%	61	24.1%	
	41–50	79	76.0%	25	24.0%	
	>50	27	100.0%	0	0.0%	
Gender	Men	59	79.7%	15	20.3%	0.911
	Women	338	79.2%	89	20.8%	
Marriage status	Currently married	323	78.0%	91	22.0%	0.141
	Currently not married	74	85.1%	13	14.9%	
Education level	< Undergraduate	117	88.6%	15	11.4%	**0.002**
	≧Undergraduate	280	75.9%	89	24.1%	
Family income	< 5,000 CYN	36	75.0%	12	25.0%	0.102
	5,000–9,999 CYN	172	83.9%	33	16.1%	
	10,000–19,999 CYN	128	79.5%	33	20.5%	
	20,000–29,999 CYN	31	68.9%	14	31.1%	
	≧30,000 CYN	30	71.4%	12	28.6%	
Working years	0–4 years	40	83.3%	8	16.7%	0.34
	5–9 years	74	81.3%	17	18.7%	
	10–19 years	182	75.8%	58	24.2%	
	≧20 years	101	82.8%	21	17.2%	
Professional title	Junior	218	85.5%	37	14.5%	**0.001**
	Medium	154	71.3%	62	28.7%	
	Senior	25	83.3%	5	16.7%	
Working place	Urban	73	67.7%	35	32.4%	**0.002**
	Suburb	169	80.1%	42	19.9%	
	Rural	155	85.2%	27	14.8%	
**Daily vaccination work**						
Daily number of vaccinations	< 100 persons	194	81.9%	43	18.1%	**0.024**
	100–199 persons	159	79.1%	42	20.9%	
	200–299 persons	36	65.5%	19	34.5%	
	≧300 persons	8	100.0%	0	0.0%	
Vaccination working days per week	0.5 day	31	75.6%	10	24.4%	0.264
	1 day	153	84.1%	29	15.9%	
	1.5–2 days	57	77.0%	17	23.0%	
	2.5–3 days	119	74.8%	40	25.2%	
	≧3.5 days	37	82.2%	8	17.8%	
**COVID-19 vaccination work**						
COVID-19 vaccination doses	0–9,999 doses	118	84.3%	22	15.7%	0.302
	10,000–19,999 doses	99	78.6%	27	21.4%	
	20,000–39,999 doses	63	74.1%	22	25.9%	
	40,000–59,999 doses	51	82.3%	11	17.7%	
	≧60,000 doses	66	75.0%	22	25.0%	
Working time	Not participating	24	80.0%	6	20.0%	**< 0.001**
	During working hours	64	88.9%	8	11.1%	
	A few of works take up time off work or rest days	248	82.9%	51	17.1%	
	Most of work takes up time off work or rest days	51	62.2%	31	37.8%	
	All the work takes up time off work or rest days	10	55.6%	8	44.4%	
**COVID-19 prevention and control work**						
Working time	Not participating	23	100.0%	0	0.0%	**< 0.001**
	During working hours	40	88.9%	5	11.1%	
	A few of works take up time off work or rest days	216	83.1%	44	16.9%	
	Most of work takes up time off work or rest days	104	69.8%	45	30.2%	
	All the work takes up time off work or rest days	14	58.3%	10	41.7%	
Participation time	Not participating	23	100.0%	0	0.0%	**0.026**
	< 1 week	19	95.0%	1	5.0%	
	1 week−1 month	55	79.7%	14	20.3%	
	1–2 months	86	80.4%	21	19.6%	
	≧2 months	214	75.9%	68	24.1%	

The bold values in the table indicate that the variables are statistically significant.

Further, the binary logistic regression model revealed that the possibility of having burnout symptoms was significantly higher in participants who had high education level (OR = 2.186, 95% CI:1.188–4.022, *p* = 0.012), medium professional title (OR = 2.095, 95% CI:1.303–3.369, *p* = 0.002), most (OR = 4.001, 95% CI:1.656–9.666, *p* = 0.002) and all (OR = 5.061, 95% CI:1.507–16.999, *p* = 0.009) of COVID-19 vaccination work takes up time off work or rest days (Table 3).

**Table 3:** Binary logistic regression results of burnout among vaccination staff.

**Variable**	***P***	**Odd ratio (OR)**	**95% Confidence interval (CI)**	
Education level				
< Undergraduate	Ref	Ref	Ref	Ref
≧Undergraduate	**0.012**	2.186	1.188	4.022
Professional title	0.006			
Junior	Ref	Ref	Ref	Ref
Medium	**0.002**	2.095	1.303	3.369
Senior	0.901	0.935	0.326	2.680
Working time of COVID-19 vaccination work	0.000			
During working hours	Ref	Ref	Ref	Ref
A few of works take up time off work or rest days	0.482	1.339	0.593	3.020
Most of work takes up time off work or rest days	**0.002**	4.001	1.656	9.666
All the work takes up time off work or rest days	**0.009**	5.061	1.507	16.999
Not participating	0.431	1.609	0.493	5.259

The bold values in the table indicate that the variables are statistically significant.

### 3.4. Factors associated with MBI-GS three components in vaccination staff

The average burnout score was 10.73 ± 6.41 on the EE subscale, 6.74 ± 5.27 on the CY subscale, and 17.95 ± 7.83 on the PA subscale. MBI-GS subscale scores after grouping according to demographics and work-related variables were present in Table 4. Univariable analysis showed that all variables for COVID-19 vaccination work and COVID-19 prevention and control work were statistically associated with EE and CY. Based on this, variables associated with CY added age, education level, working years, professional title, working place, and vaccination working days per week. Compared with CY, EE added the statistically significant variables of family income and the daily number of vaccinations. Regarding PA, only age, working years, professional title, and participation time for COVID-19 prevention and control were statistically significant (*P* < 0.1) (Table 4).

**Table 4:** MBI-GS subscale scores in grouped demographics and work-related variables.

**Variables**		**EE**			**CY**			**PA**		
		***x*** ±***s***	**F/t**	***P***	***x*** ±***s***	**F/t**	***P***	***x*** ±***s***	**F/t**	***P***
**Sociodemographic characteristics**										
Age	18–30	9.17 ± 6.08	5.553	**0.001**	5.86 ± 4.90	2.884	**0.035**	16.52 ± 7.68	3.759	**0.011**
	31–40	22.09 ± 6.53			7.08 ± 5.38			17.76 ± 7.54		
	41–50	12.17 ± 6.60			7.37 ± 5.53			19.99 ± 7.98		
	>50	8.52 ± 3.76			5.04 ± 3.93			17.96 ± 9.36		
Gender	Men	9.96 ± 6.16	1.250	0.264	6.18 ± 5.10	1.006	0.316	17.68 ± 8.91	0.103	0.748
	Women	10.86 ± 6.45			6.84 ± 5.30			17.99 ± 7.64		
Marriage status	Currently married	10.91 ± 6.52	1.980	0.160	6.77 ± 5.31	0.046	0.830	18.06 ± 7.90	0.529	0.467
	Currently not married	9.85 ± 5.80			6.63 ± 5.10			17.39 ± 7.53		
Education level	< Undergraduate	9.21 ± 5.48	10.221	**0.001**	5.92 ± 4.73	4.437	**0.036**	17.39 ± 7.94	0.915	0.339
	≧Undergraduate	11.27 ± 6.63			7.04 ± 5.42			18.15 ± 7.80		
Family income	< 5,000 CYN	11.77 ± 7.13	2.225	**0.065**	7.25 ± 5.31	1.425	0.224	17.65 ± 7.77	1.399	0.233
	5,000–9,999 CYN	9.80 ± 6.05			6.11 ± 4.85			17.13 ± 7.66		
	10,000–19,999 CYN	10.99 ± 6.34			6.98 ± 5.42			18.42 ± 7.69		
	20,000–29,999 CYN	12.13 ± 7.30			7.53 ± 6.03			19.78 ± 8.30		
	≧30,000 CYN	11.60 ± 6.14			7.52 ± 5.60			18.50 ± 7.80		
Working years	0–4 years	8.81 ± 6.32	3.295	**0.020**	5.17 ± 4.83	2.154	**0.093**	17.60 ± 9.23	3.758	**0.011**
	5–9 years	9.64 ± 6.65			6.27 ± 5.70			15.95 ± 7.06		
	10–19 years	11.11 ± 6.37			7.09 ± 5.18			17.96 ± 7.38		
	≧20 years	11.54 ± 6.14			7.03 ± 5.18			19.54 ± 8.38		
Professional title	Senior	12.10 ± 5.03	16.182	**<** **0.001**	7.17 ± 4.22	14.179	**<** **0.001**	21.30 ± 7.80	6.333	**0.002**
	Medium	12.37 ± 6.71			8.08 ± 5.70			18.74 ± 7.64		
	Junior	9.18 ± 5.91			5.56 ± 4.71			16.88 ± 7.84		
Working place	Urban	12.88 ± 7.22	12.888	**<** **0.001**	8.32 ± 6.11	8.616	**<** **0.001**	17.73 ± 8.04	0.402	0.669
	Suburb	11.04 ± 5.86			6.82 ± 4.86			18.31 ± 7.30		
	Rural	9.09 ± 6.10			5.71 ± 4.50			17.65 ± 8.32		
**Daily vaccination work**										
Daily number of vaccinations	< 100 persons	9.69 ± 6.61	4.328	**0.005**	6.24 ± 5.23	1.783	0.149	17.75 ± 7.89	0.685	0.562
	100–199 persons	11.65 ± 5.94			7.10 ± 5.29			18.17 ± 7.65		
	200–299 persons	12.00 ± 6.89			7.75 ± 5.46			17.47 ± 8.15		
	≧300 persons	9.63 ± 3.07			5.88 ± 2.95			21.38 ± 8.91		
Vaccination working days per week	0.5 day	11.02 ± 5.40	3.548	**0.007**	6.88 ± 4.42	2.918	**0.021**	18.05 ± 6.99	0.585	0.673
	1 day	9.45 ± 6.29			5.80 ± 5.12			17.65 ± 7.88		
	1.5–2 days	10.93 ± 5.39			6.91 ± 4.63			18.11 ± 7.07		
	2.5–3 days	12.02 ± 6.83			7.73 ± 5.61			18.53 ± 8.37		
	≧3.5 days	10.76 ± 7.00			6.67 ± 5.80			16.69 ± 7.74		
**COVID-19 vaccination work**										
COVID-19 vaccination doses	0–9,999 doses	9.63 ± 5.58	4.171	**0.002**	6.05 ± 4.78	3.902	**0.004**	17.63 ± 7.44	0.328	0.859
	10,000–19,999 doses	10.17 ± 6.65			6.04 ± 5.10			18.29 ± 7.94		
	20,000–39,999 doses	12.64 ± 6.89			8.41 ± 6.19			17.73 ± 7.77		
	40,000–59,999 doses	10.06 ± 6.38			6.39 ± 5.30			17.42 ± 8.99		
	≧60,000 doses				7.49 ± 4.89			18.53 ± 7.59		
Working time	Not participating	9.70 ± 6.51	16.276	**<** **0.001**	6.03 ± 5.45	10.578	**<** **0.001**	18.57 ± 6.922	0.254	0.907
	During working hours	7.83 ± 5.69			4.74 ± 4.49			18.15 ± 8.88		
	A few of works take up time off work or rest days	10.17 ± 5.75			6.38 ± 5.06			17.67 ± 7.91		
	Most of work takes up time off work or rest days	14.89 ± 6.31			9.55 ± 5.00			18.50 ± 6.93		
	All the work takes up time off work or rest days	14.33 ± 9.45			9.11 ± 7.05			18.11 ± 7.99		
**COVID-19 prevention and control work**										
Working time	Not participating	5.30 ± 3.94	18.645	**<** **0.001**	2.52 ± 3.29	13.536	**<** **0.001**	19.83 ± 11.52	0.748	0.559
	During working hours	7.98 ± 6.31			4.80 ± 5.15			18.11 ± 8.26		
	A few of works take up time off work or rest days	9.82 ± 5.92			6.13 ± 5.05			17.45 ± 7.67		
	Most of work takes up time off work or rest days	13.22 ± 6.09			8.71 ± 5.09			18.42 ± 7.24		
	All the work takes up time off work or rest days	15.42 ± 7.25			8.88 ± 5.40			18.33 ± 8.26		
Participation time	Not participating	5.30 ± 3.94	7.006	**<** **0.001**	2.52 ± 3.29	5.123	**<** **0.001**	19.83 ± 11.52	2.266	**0.061**
	< 1 week	7.20 ± 4.60			5.10 ± 3.63			14.60 ± 8.18		
	1 week−1 month	10.72 ± 6.92			7.22 ± 5.36			17.10 ± 7.13		
	1–2 months	10.52 ± 5.85			6.43 ± 4.86			17.06 ± 7.38		
	≧2 months	11.50 ± 6.49			7.21 ± 5.46			18.57 ± 7.71		

The bold values in the table indicate that the variables are statistically significant.

**Figure 3:**
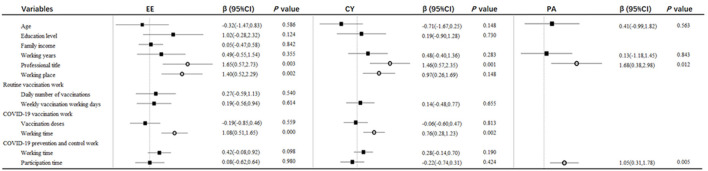
Multivariable analysis of the risk factors for EE, CY, and PA among vaccination staff.

Then multiple linear regressions were performed to identify independent related factors to each MBI-GS subscale. EE was independently correlated with professional title (β = 1.647, t = 2.998, *p* = 0.003), working place (β = 1.403, t = 3.108, *p* = 0.002), working time for COVID-19 vaccination (β = 1.079, t = 3.717, *p* < 0.001). CY was independently correlated with professional title (β = 1.460, t = 3.216, *p* = 0.001), working place (β = 0.971, t = 2.671, *p* = 0.008), working time for COVID-19 vaccination (β = 0.755, t = 3.119, *p* = 0.002). PA was independently correlated with professional title (β = 1.677, t = 2.534, *p* = 0.012) and participation time for COVID-19 prevention and control work (β = 1.047, t = 2.804, *p* = 0.005) (Figure 3).

## 4. Discussion

During the COVID-19 pandemic, the work of COVID-19 vaccination and epidemic control has greatly increased the workload of vaccination staff, therefor it is necessary to investigate the burnout situation of vaccination staff. The main findings of this study were: (1) The overall prevalence of burnout syndrome among vaccination staff was 20.8% in Hangzhou, China. (2) The predictors associated with job burnout were educational level, professional title, and COVID-19 vaccination working time. (3) The vaccination staff was experiencing a high degree of exhaustive emotion, cynicism, and especially low personal accomplishment.

As far as we know, there has not been much consensus on the “diagnosing” of burnout. First, different criteria were used to distinguish the high and low levels of the three dimensions. For example, studies used 9 and 13 as the cutoff to distinguish the different levels of EE (19). However, other studies used 11 and 15 (42, 43) or 11 and 14 (44). Second, the criteria for determining burnout are inconsistent. Studies used the three components' weighted score as criteria (44–46), and other studies used any of the components to classify the level of burnout (43, 47). In this study, referring to Huo et al. (19) and Li et al.'s (48) studies, we used the “exhaustion+1” criterion to define burnout symptoms and distinguish different levels of burnout. Brenninkmeijer et al. indicated that a categorization in which both high exhaustion and high distance or low competence were conditions for burnout (“exhaustion+1”), resulted in a relatively small chance of an inaccurate qualification of burnout and seemed to be an effective categorization for mapping differences in burnout (42).

The results of this study showed that vaccination staff had a high level of burnout (20.8%), and the prevalence of EE, CY, and PA at high in this study was 26.7, 21.4, and 50.7%, respectively. Compared to previous studies using the same criterion, the level of burnout in vaccination staff was lower than that in medical staff (36.5%) (19). The high level of EE and CY in vaccination staff was also lower than that in medical staff (EE: 40.9%, CY: 63.7%) and frontline health professionals (EE: 34.2%, CY: 50.8%), respectively (19, 48). Based on this, it could be assumed that the situation of job burnout, EE and CY for vaccination staff was better than that for other medical staff during the COVID-19 epidemic in China. Exhaustion emotion is the central quality of burnout and is associated with workload, including working hours (49, 50), work shifts (51), and work pressure (52). Compared with vaccination staff, other medical workers, especially the frontline health professionals (48), had a heavy workload to save and care for COVID-19 patients, and they were under tremendous pressure, such as the high risk of contracting the virus and bringing to their families (52). All of this could cause them to have higher levels of EE. Cynicism emerged from the presence of work overload and social conflict. It prompted medical staff to take action to distance themselves emotionally and cognitively from their work. Previous studies have indicated that deteriorating doctor-patient relationships could lead to a high level of CY in medical staff (53, 54). In China, the doctor-patient relationship has always been a big problem (55, 56). According to previous studies, difficulty in seeing a doctor, poor communication, high medical expenses, and high expectations for doctors were all the influencing factors for bad doctor-patient relationships (18). These conditions were more common in medical staff in hospitals than in vaccination clinics. Therefore, we hypothesized that these factors lead to higher levels of EE and CY in medical workers than in vaccination staff. However, on the contrary, regarding the low level of PA, the situation is much worse in the vaccination staff than in other medical staff. From Guo and Li's study, the level of PA at low in medical staff was 35.2 and 46%, respectively (19, 48), which was lower than that in vaccination staff (50.7%) in this study. The component of PA represents the self-evaluation dimension of burnout and refers to feelings of incompetence and a lack of achievement and productivity in work (57). First, vaccination staff is public health providers working in primary care institutions. In China, the social status of primary medical institutions is generally lower than that of hospitals. People are more willing to bypass primary medical institutions to seek care at hospitals (58). Similarly, public health providers have a lower social status than clinicians. People trust clinicians more than public health providers. All these factors contributed to the low PA of vaccination staff (59). Second, for the work of vaccination staff, on the one hand, the main work was to vaccinate the population. Their sense of job accomplishment was not as apparent as doctors treating patients and saving lives. On the other hand, vaccine hesitancy is widespread in the population (60–62). Vaccination staff who regularly interact with vaccine-hesitant people was prone to question their competence and had a higher level of burnout and lower level of job satisfaction (63), which could lead them to doubt the value of their work. In addition, during the COVID-19 period, like clinicians, vaccination staff made an outstanding contribution to the fight against the COVID-19 epidemic. However, compared with clinicians and other medical workers, vaccination staff had low income, low returns, low social status, and low social support (64). All these reasons contributed to the low level of PA in vaccination staff (26). In the future, more studies are needed to study the interventions to reduce the PA in vaccination staff.

In this study, the score of the three components of EE, CY, and PA were 10.73 ± 6.41, 6.74 ± 5.27, and 17.95 ± 7.83, respectively. According to previous studies, during the COVID-19 epidemic in China, vaccination staff had lower scores of EE and CY but higher scores of PA than other health professionals (19, 48, 65, 66). The results were consistent with the distribution of high levels of EE, CY, and low levels of PA in vaccination staff and medical staff discussed above. However, it was worth mentioning that although the EE and CY scores of vaccination staff were lower than those of medical workers, it did not mean that the EE and CY levels of vaccination staff were not high. To Lu's study, the scores of EE and CY in biosafety laboratory staff were 10.00 ± 5.99 and 4.64 ± 4.59, which were lower than that in vaccination staff during the COVID-19 epidemic (52). With the arrival of COVID-19, the workload of vaccination staff has dramatically increased. In addition to routine work of vaccinations for children and some adults, they also needed to vaccinate people over the age of three. This study found that nearly 80% of participants reported that the COVID-19 vaccination work took up time off work and rest days. Furthermore, the vaccination staff was involved in the COVID-19 prevention and control work. They need to concrete implementation of COVID-19 prevention and control. Figure 1 shows that 88.2% of participants worked for nucleic acid sampling in the community, 75.8% worked for nucleic acid sampling for home quarantine, and 86.4% of participants in this study reported that the COVID-19 prevention and control work took up time off work and rest days. As a result, the workload and working hours for vaccination staff had increased significantly, which caused the high level of EE. After that, vaccination staff became indifferent and repulsive to their service objects and to their own profession, thus causing a high level of CY (23). On the other hand, as we know, there may be an tiny chance of adverse events following vaccination. The amount of COVID-19 vaccine inoculated is enormous. Therefore, the number of people with adverse events becomes obvious in public view. Some people attributed the adverse events to vaccination staff and even attached violence to them. This would worsen the working environment of vaccination staff and cause high CY. To better understand the level of EE, CY, and PA among vaccination staff and to compare them with other health care workers, further work is required to establish a norm for medical workers and to monitor the job burnout level of vaccination staff in a long-term manner.

Among the related factors of job burnout, we found that vaccination staff with higher education level had more job burnout than those with lower education level. This was consistent with previous findings studied in medical staff (67–69). A possible explanation for this might be that highly educated vaccination staff usually had more responsibility and expectations (67). They would have a more important role played in work, which pushed them to suffer from a greater risk of job burnout (52).

Another finding was that vaccination staff with the medium professional title had a higher level of burnout, EE, and CY. Previous studies also reported this finding in primary healthcare workers and nurses in China (45, 70). There were several possible explanations for this result. First, according to China's medical system and the professional title system of health professionals (71), vaccination staff with medium titles were always in middle age and the central workforce in vaccination clinics, during which the heavy workload might result in a high level of EE (72). Second, vaccination staff with medium professional titles were in the promotion period of careers. However, in China, the work resource for health care workers is very scarce (45, 64). Only a tiny percentage of vaccination staff with medium professional titles could upgrade to senior professional titles (73), which inevitably leading to competition among colleagues. The lack of critical resources and the poor quality of colleague relationships would reduce job satisfaction and increase CY in vaccination staff (24). In terms of PA, a possible explanation might be that with the rise of professional title, the workability and work sense of accomplishment of vaccination staff were also gradually increased, and they were more able to appreciate their personal and work value.

The result of this study showed that working place was associated with EE and CY. The EE and CY scores of vaccination staff were highest in urban areas and lowest in rural areas. Related conclusions from previous studies were mixed. A general practitioner study showed no difference in EE, CY, and PA between urban and rural areas (74). Another study showed that compared with rural areas, public health service providers in urban areas had higher EE and CY but no statistical difference in PA (59). Within the context of our study setting, there were several possible explanations for the finding in this study. First, in Hangzhou, vaccination-related work has been done better in urban areas than in suburban or rural areas. Vaccination staff in urban areas have higher requirements for their work, such as a higher vaccination rate, better service attitude, and a more convenient service experience. These might lead to an increase in workload, and increase their working pressure. Previous literature had reported an association between working pressure and burnout (20). Secondly, the massive influx of migrants in urban areas has brought considerable challenges to the COVID-19 prevention and control efforts, making COVID-19 epidemic prevention and control more difficult (75). Thirdly, compared with urban areas, rural or suburban areas had relatively better health care environments and better doctor-patient relationships (76). All these factors might cause result in high EE and CY in the urban area.

Regarding the job-related factors, we found that vaccination staff who reported that the work of COVID-19 vaccination took up more time off work or rest days was more likely to be burnout and have a high level of EE and CY. The more work that takes up time off work or rest days, the longer work hours will be. Moreover, the relationship between prolonged working hours and burnout, EE, and CY has been well demonstrated (49, 54, 67, 77). Considering that COVID-19 vaccination is currently a positive and effective way to prevent COVID-19 (78), and booster shots of COVID-19 vaccine might be needed in the future (35), it is essential to improve the efficiency of COVID-19 vaccination and arrange working hours reasonably to reduce the job burnout among vaccination staff.

The current study found that the longer time vaccination staff participated in COVID-19 prevention and control, the more personal accomplishment they felt. Since 2022, there have been multiple COVID-19 outbreaks in Hangzhou. The vaccination staff was involved in the COVID-19 prevention and control work, including nucleic acid sampling, extermination, and hospital transmission, et al. (Table 1). Through the joint efforts of vaccination staff and the whole society, the epidemic in Hangzhou has been controlled at a stable level (79), which might give vaccination staff a great sense of accomplishment and work value. Furthermore, vaccination staff who participated in COVID-19 prevention and control work might get more honors, more bonuses, and higher social support from superior and organization, which could improve their PA.

This study has strengths and limitations. To our knowledge, this is the first study to investigate burnout among vaccination staff in China. The three main aspects of work for vaccination staff during COVID-19, including routine vaccination work, the COVID-19 vaccination work, and the COVID-19 prevention and control work, were all considered in this study. However, this study has some limitations. First, there is no consensus on the diagnosis of job burnout. We only selected one of the diagnosis methods, so it was difficult to directly compare the prevalence of job burnout with other studies. Second, the indicators of workload in this paper were not very precise. We could not determine the amount of vaccination for each vaccination staff, so the vaccination dose for each vaccination staff's clinic was used. In addition, regarding working hours, we used the subjective judgment method of vaccination staff' self-assessment, which may be biased compared to the specific assessment time. It was better to use concrete numbers, i.e., 40 h per week, to measure burnout. Third, because this survey was conducted by online questionnaire, compared with a face-to-face questionnaire survey, it was inevitable that there would be some problems with survey quality, such as unclear questionnaire questions and filling errors.

## 5. Conclusion

The present study found that vaccination staff in Hangzhou, China, had high levels of job burnout, EE and CY, and these conditions were better than than other medical staff The level of PA among vaccination staff was much worse than other medical staff. The factors influencing burnout included level of education, professional title, and working time for COVID-19 vaccination work. The professional title, working place, and the working time for COVID-19 vaccination were associated with the degree of EE and CY. For PA, the associated factors were professional title and participation time for COVID-19 prevention and control. Interventions should be taken to reduce the level of job burnout and alleviate psychological pressure in vaccination staff, especially to enhance their personal achievement. Further research should conduct to reach consensus on the “diagnosing” of burnout, and the research on the norm of burnout among medical staff is warranted.

## Data availability statement

The datasets presented in this article are not readily available because the data that support the findings of this study are available from the corresponding author upon reasonable request. Requests to access the datasets should be directed to YL, smileforever81@126.com.

## Ethics statement

The studies involving human participants were reviewed and approved by Ethics Committee of the Hangzhou Center for Disease Control and Prevention. The patients/participants provided their written informed consent to participate in this study.

## Author contributions

WG and YL planned and designed the study. ZL, YX, XuZ, and JC were responsible for data management. JuW, XC, and JiW for data analysis. WG drafted the manuscript. JD and XiZ for supervision. All authors contributed to interpretation of study results, critical revision of the paper and approval of final version, and agree to be accountable for all aspects of this article.
